# Imidazole propionate in type 2 diabetes mellitus and cardiovascular diseases: a mini review

**DOI:** 10.3389/fimmu.2024.1454210

**Published:** 2024-08-29

**Authors:** Qian Xu, Wenting Wang, Yiwen Li, Yanfei Liu, Yue Liu

**Affiliations:** ^1^ National Clinical Research Center for Chinese Medicine Cardiology, Xiyuan Hospital, Chinese Academy of Chinese Medical Sciences, Beijing, China; ^2^ The Second Department of Gerontology, Xiyuan Hospital, China Academy of Chinese Medical Sciences, Beijing, China

**Keywords:** microbiota metabolite, imidazole propionate, type 2 diabetes mellitus, cardiovascular disease, research progress

## Abstract

Oral and gut microbiota can interact with the host by producing a diverse range of bioactive metabolites, thereby influencing overall host health. Imidazole propionate (ImP), a histidine-derived metabolite produced by microbes associated with diabetes mellitus, has attracted considerable attention on account of its roles in metabolic and cardiovascular diseases. In this article, we review the metabolic pathways of ImP, as well as its roles and therapeutic potential in type 2 diabetes mellitus and cardiovascular diseases. Future research should focus on key enzymes and regulatory factors in the ImP metabolic pathway, interactions with other metabolites, and conduct large-scale clinical studies to gain a more comprehensive understanding of the role of ImP in diverse populations and disease contexts. Moreover, targeted interventions against ImP could provide novel strategies for preventing and treating metabolic and cardiovascular diseases.

## 1 Background

The human body hosts a diverse range of dynamic and balanced microbial communities, the homeostasis of which plays a pivotal role in maintaining human health. With continuous advances in the development of molecular tools and technologies, our understanding of the microbiota has progressively deepened. These microorganisms, as key participants, interact with the host by producing diverse bioactive metabolites, thereby influencing host metabolic health. Additionally, they are closely associated with immune, energy, lipid, and glucose metabolic pathways (1). Researchers using advanced metabolomic tools, such as ultra-performance liquid chromatography-tandem mass spectrometry (UPLC-MS/MS), have identified several key metabolites, including bile acids (2), short-chain fatty acids (3), branched-chain amino acids (4), and trimethylamine N-oxide (5). These metabolites can induce a series of physiological and pathological changes in the host, thereby having pronounced effects on energy metabolism, nutrient absorption, and microbiota composition. In healthy states, microbial metabolites contribute to the maintenance of essential host functions. Conversely, imbalances in microbial community composition are associated with a range of diseases, including metabolic diseases, cardiovascular diseases, gastrointestinal diseases, neurodegenerative diseases, and cancer (6). Imidazole propionate (ImP), a metabolite newly identified in diabetes-related dysbiosis, is gaining increasing attention (7). This review outlines the progress in ImP research to explore its physiological functions and highlight its clinical potential.

## 2 Metabolic pathway of ImP

Diet influences the structure of the host microbiota and provides substrates for microbial enzymes during digestion, generating metabolites that regulate host physiological functions. Histidine (His) is an essential dietary amino acid that cannot be synthesized within the body and thus must be obtained via dietary sources (8). The primary pathway for His catabolism begins with deamination catalyzed by histidase, subsequently producing urocanate and ammonia (9). His plays vital roles in erythropoiesis, hemoglobin metabolism, and skin repair, and has been demonstrated to enhance insulin sensitivity, reduce oxidative stress, and lower plasma inflammatory markers (10).

**Figure 1:**
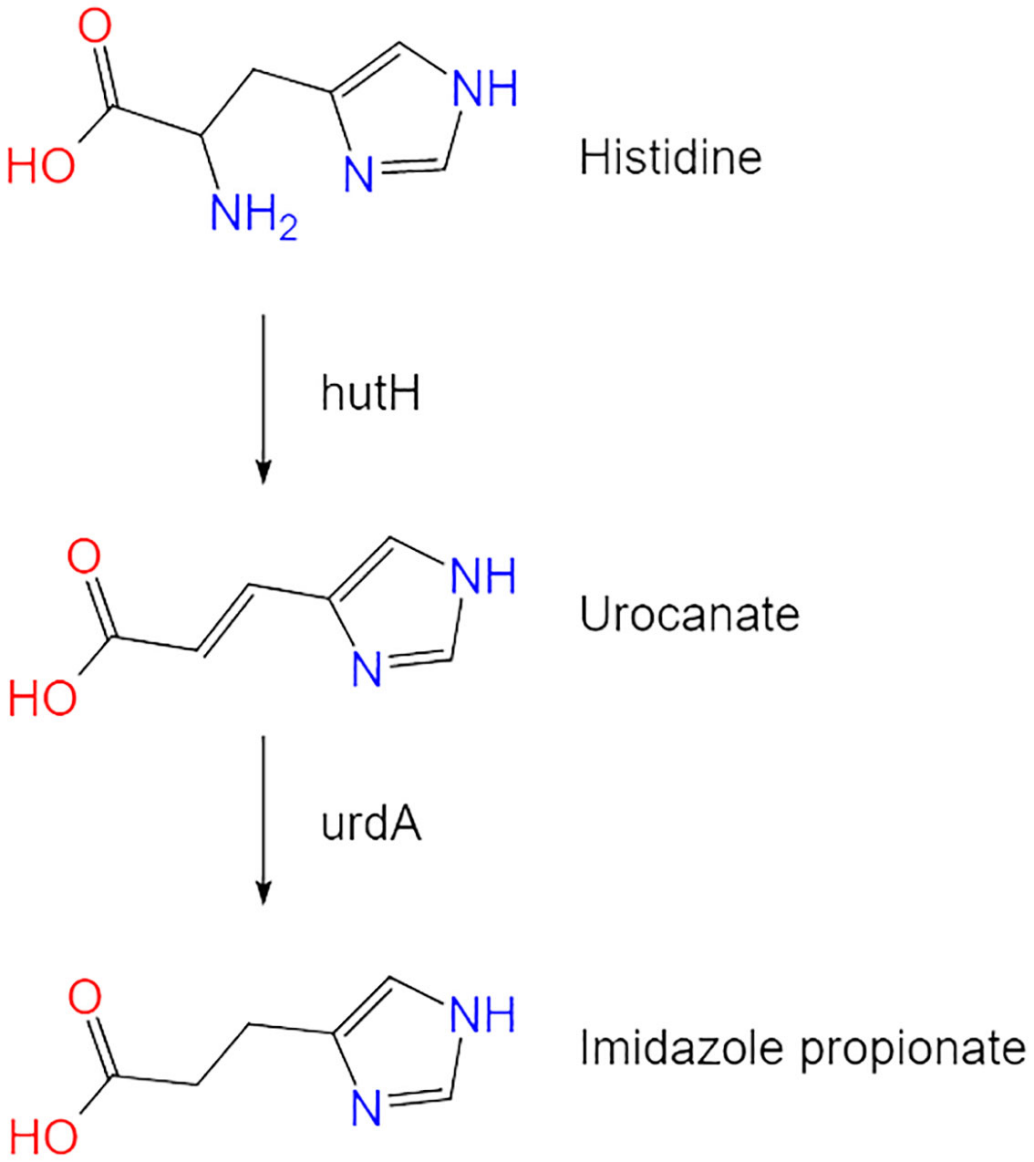
The metabolic pathway of imidazole propionate. Histidine is converted to imidazole propionate through the action of key enzymes hutH and UrdA.

ImP is a His-derived metabolite produced by microbes associated with diabetes. Its metabolic pathways are mediated by multiple bacterial groups and microbial enzymes. Histidine ammonia-lyase (hutH) and urocanate reductase (UrdA) are key enzymes responsible for converting His to ImP. The synthesis of ImP is primarily dependent on the activity of the bacterial enzyme UrdA, which catalyzes the reduction of urocanate to ImP (7). By unidirectionally reducing urocanate to ImP, this enzyme provides an alternative pathway for His metabolism (10, 11) (**Figure 1**).

Although ImP is a microbially dependent metabolite, the specific bacterial communities involved in its production have yet to be comprehensively elucidated. Dysbiosis among oral–gut microbiota may provide an environment favorable for ImP production (12). Potential ImP producers include *Streptococcus mutans*, *Lactobacillus paraplantarum*, *Eggerthella lenta*, *Clostridium symbiosum*, *Clostridium scindens*, *Clostridium bolteae*, *Pseudoflavonifractor*, *Eubacterium eligens*, *Ruminococcus gnavus*, and *Veillonella* (13–16). Moreover, some ImP-associated species, such as *Blautia hansenii*, *Bifidobacterium reuteri*, and *Veillonella parvul*, are also positively correlated with hutH expression (17).

## 3 ImP and diseases

### 3.1 ImP and type 2 diabetes mellitus

The microbiota, acting as an intermediary between the host and diet, can produce various metabolites that, after further modification by host enzymes, can promote the progression of metabolic diseases. ImP was first discovered in patients with intestinal inflammation in 1970 (18). Subsequent research has indicated that ImP can induce intestinal inflammation, impair the intestinal barrier, and influence goblet cell proliferation (19). In recent years, Koh et al. have focused on ImP research and found elevated levels of ImP in diabetic patients. They discovered that ImP activates p38γ, promotes p62 phosphorylation, and subsequently activates mTORC1. Through the p38γ/p62/mTORC1 pathway, ImP inhibits the insulin receptor substrate (IRS), leading to insulin resistance (IR) and inducing glucose metabolism disorders (7, 14). Additionally, ImP has been found to inhibit metformin-induced AMPK activation via p38γ and Akt, thereby reducing the response to the hypoglycemic effects of metformin. Higher levels of ImP have been observed in type 2 diabetes mellitus receiving metformin treatment, while the average levels of the ImP precursor urocanate are similar in both groups (20). Animal studies have demonstrated that intraperitoneal injection of ImP can promote these effects, suggesting that ImP directly influences the development of metabolic diseases. Levels of ImP are positively correlated with the concentrations of glycated hemoglobin, blood glucose, insulin, and HOMA-IR, and negatively associated with HOMA-β. Similarly elevated levels of ImP are also associated with increases in glucose, insulin, and C-peptide levels 2 h after an oral glucose tolerance test (14). Notably, ImP levels do not appear to be directly related to dietary His intake, but are associated with unhealthy diets low in fiber and unsaturated fatty acids, leading to increases in populations of ImP-producing microbiota. Consistently, a high intake of saturated fats (e.g., a high cheese intake) has been observed to be positively correlated with ImP levels, whereas high intakes of fiber and unsaturated fat (typically from vegetables and nuts) was found to be associated with a negative correlation. Healthier dietary patterns are thus associated with lower ImP levels (14).

A single-center, prospective, cross-sectional study showed that diabetic patients with abnormal stool consistency had higher levels of ImP compared to those with normal stool consistency. This difference was attributed to respective differences in bile acid ratios and fecal microbiota structure (21). ImP has been reported to inhibit angiogenesis in diabetic wound healing by suppressing SPNS2-mediated S1P secretion and activating the Rho signaling pathway (22). In diabetic nephropathy (DN) patients, significantly elevated levels of ImP have been observed, which are positively correlated with the urinary albumin/creatinine ratio (uACR) and negatively correlated with the estimated glomerular filtration rate (eGFR), suggesting that ImP may contribute to promoting DN progression. Animal experiments have shown that intraperitoneal injection of ImP can directly promote renal interstitial fibrosis in DN mice. Additionally, ImP-treated human renal proximal tubular (HK-2) cells were found to be characterized by increases in the expression of fibrosis-related proteins. Further research indicated that ImP targets Hsp90α, thereby activating the PI3K-Akt signaling pathway, and inducing epithelial-mesenchymal transition(EMT) in renal tubular epithelial cells, and hence promoting glomerular interstitial fibrosis (23). ImP has also been identified as a potential inducer of steatosis and liver inflammation (24). For example, in a non-alcoholic steatohepatitis (NASH) animal model, Göttingen minipigs fed a choline-deficient, L-amino acid-defined, high-fat diet (CDAHFD) for 8 weeks exhibited changes in colonic microbiota. These changes promoted ImP production. This response was associated with impaired hepatic insulin signaling, hyperglucagonemia, reduced glucagon receptor expression, and disruption of the liver-α cell axis (12).

These studies indicate that ImP not only plays a significant role in glucose metabolism disorders but is also closely associated with metabolic diseases. Its levels are influenced by dietary patterns and microbiota dysbiosis. Therefore, the mechanisms and influencing factors of ImP in metabolic diseases deserve further in-depth research.

### 3.2 ImP and cardiovascular disease

The composition of the microbiota has also been established to be closely linked to the development of cardiovascular diseases. High levels of ImP-producing bacteria may increase the levels of circulating plasma ImP, as well as promoting atherosclerosis and cardiovascular disease by regulating host immune activation and inflammation (17). Research has shown that in overweight/obese individuals without diabetes, plasma ImP concentrations are positively correlated with diastolic blood pressure but are not associated with insulin sensitivity (25). Additionally, research has indicated that in HIV patients, ImP is associated with carotid atherosclerosis (17). Similarly, in HIV individuals, ImP levels have been shown to be related to gut microbiota dysbiosis and are significantly elevated in those with obstructive coronary artery disease (CAD), with specific bacterial groups related to ImP production showing high internal validity in these patients. Furthermore, ImP levels have been demonstrated to increase concomitantly with the degree of dysbiosis, indicating that changes in the microbiota associated with CAD may contribute to promoting elevated levels of circulating ImP (15).

A cross-sectional study of 8,973 participants without apparent atherosclerotic disease revealed that some species associated with coronary artery calcification scores were negatively correlated with indolepropionic acid but positively correlated with secondary bile acids and ImP (26). Furthermore, a study on chronic heart failure (CHF) revealed that baseline levels of ImP in a Chinese cohort were three times higher than those in a Swedish cohort, with ImP levels in the Chinese cohort increasing by 1.1- to 1.6-fold for each additional CHF comorbidity. Moreover, ImP levels were found to be higher in individuals with HFrEF and HFmEF, and lower in those with HFpEF (27). Another study indicated that ImP levels were significantly higher in CHF patients than in healthy controls, with higher ImP levels associated with more severe dysbiosis. A weak but significant negative correlation between ImP and dietary fiber was also observed. Furthermore, it was found that ImP levels were correlated with C-reactive protein and soluble CD14 levels, both of which are considered reliable markers of systemic inflammation and monocyte activation (16). Research indicates that elevated levels of ImP in heart failure patients are not only significant but also effective indicators of overall survival, independently predicting the 5-year mortality risk (28).

Collectively, these findings indicate that microbiota dysbiosis and the consequent increase in ImP levels may play important roles in the development of cardiovascular diseases. As such, ImP is not only a potential biomarker for cardiovascular risk but also an important target for future therapeutic interventions.

### 3.3 ImP and other diseases

Research conducted to date has provided evidence that bacterial vaginosis-related epithelial disruption and mucosal inflammation are associated with the mTOR pathway and specific metabolites such as ImP. *In vitro* experiments have further confirmed that ImP can directly influence epithelial barrier function and activate the mTOR pathway (29). Although ImP is typically considered an undesirable microbial metabolite, it has been demonstrated that ImP treatment can significantly alleviate clinical symptoms in mice with atopic dermatitis-like skin lesions, including ear thickness, epidermal and dermal thicknesses, and IgE levels (30). Interestingly, animal experiments have also shown that oral administration of ImP can mitigate radiation-induced cardiopulmonary toxicity, thus indicating that ImP may be a key secondary metabolite through which His counters radiation damage (31). These findings suggest that the role of ImP in different pathological contexts is complex and diverse, thereby highlighting the necessity for further in-depth research to fully elucidate its function and potential therapeutic value in the treatment of diseases.

**Figure 2:**
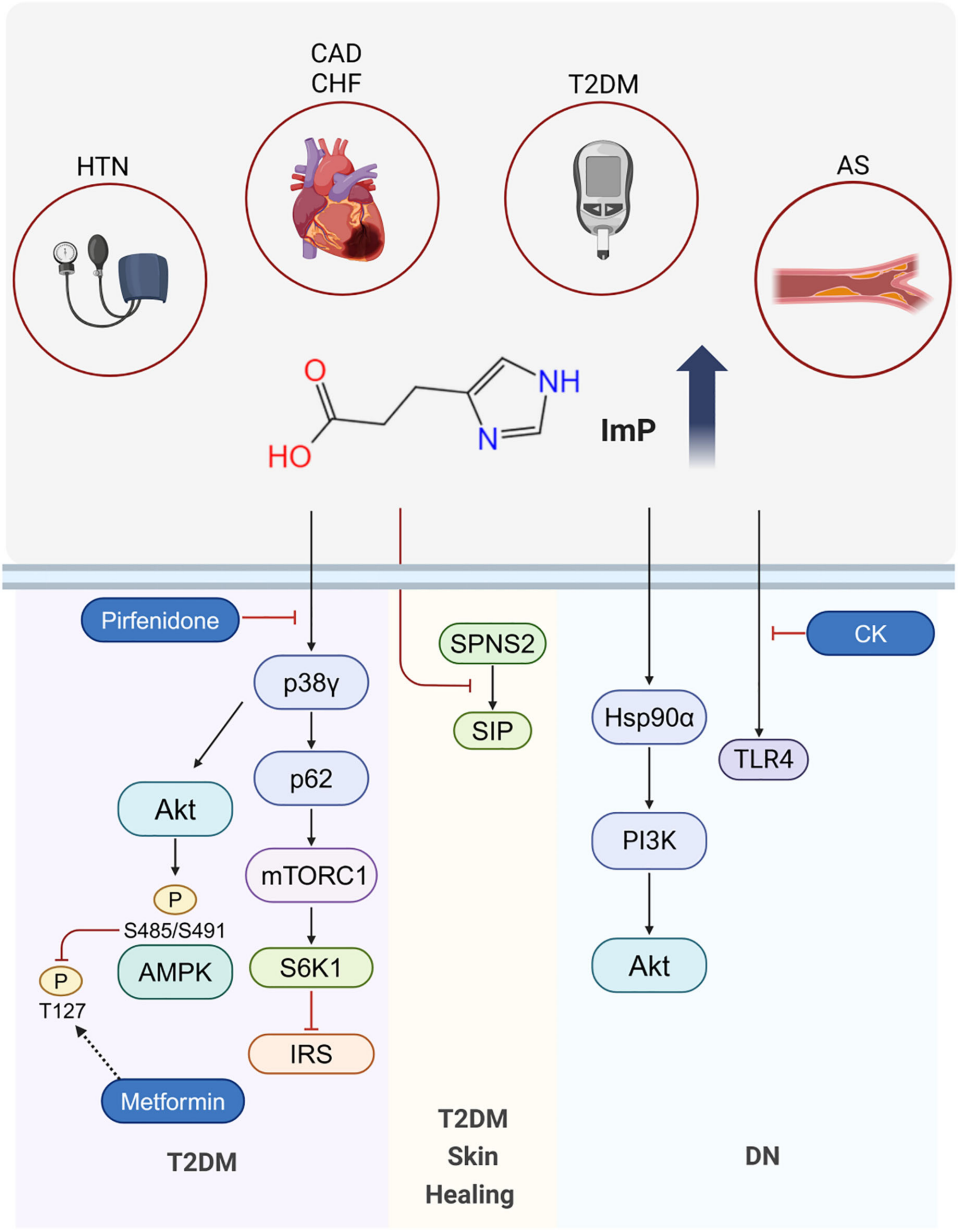
Mechanisms of ImP in Disease. ImP levels are elevated in T2DM, HTN, AS, CAD and CHF. In T2DM, ImP contributes to insulin resistance via the p38γ/p62/mTORC1 pathway and inhibits metformin-induced AMPK activation through p38γ and Akt, thereby reducing the hypoglycemic efficacy of metformin. Pirfenidone counteracts ImP-induced p38γ activation, overcoming ImP-associated metformin resistance. In T2DM-related skin healing, ImP impairs the process by suppressing SPNS2-mediated S1P secretion. In DN, ImP targets Hsp90α and activates the PI3K-Akt pathway, leading to renal fibrosis. CK ameliorates DN by inhibiting TLR4 activation.

To summarize the roles of ImP in type 2 diabetes mellitus and cardiovascular diseases, we have outlined the key pathways and mechanisms in the following figure (**Figure 2**).

## 4 Potential therapeutic approaches involving ImP

It has been demonstrated that the ginsenoside compound K (CK) can remodel the gut microbiota by reducing the abundances of *Bacteroides* and *Paraprevotella*, while promoting increases in those of *Lactobacillus* and *Akkermansia*, thereby contributing to a lowering of the serum levels of the His-derived microbial metabolite ImP. Further experiments indicated that by inhibiting ImP-induced TLR4 activation, CK alleviates the progression of diabetic nephropathy (32). Additionally, pirfenidone (a drug used to treat idiopathic pulmonary fibrosis) was found to potentially compete with ImP for the p38γ-ATP binding pocket, consequently blocking the ImP-induced activation of p38γ and leading to downstream AMPK activation to overcome ImP-associated metformin resistance (20). Nevertheless, further research is required to determine if this drug can effectively block ImP activity. Moreover, it has been demonstrated that adhering to healthier dietary habits not only contributes to reducing circulating ImP levels but also provides comprehensive cardiometabolic benefits (33). Collectively, the findings of these studies indicate that targeting ImP and its related signaling pathways may offer novel therapeutic approaches for the treatment of diverse diseases. Future research should continue to examine the specific mechanisms and clinical potential of these therapeutic strategies, focusing on their efficacy and safety in different disease contexts and populations.

## 5 Conclusion and perspective

Although research on ImP has made important progress, certain key questions remain unanswered. Firstly, the identities of the core microbiota, key enzymes, and regulatory factors in the ImP metabolic pathway have yet to be fully elucidated. Gaining a more thorough understanding of these key elements will enable us to better comprehend the metabolic processes of ImP and develop more effective intervention strategies. Secondly, the interactions between ImP and other metabolites is an important area of research that to date has received comparatively little attention. For example, whether ImP interacts with other microbial metabolites and how these interactions might influence the metabolic state of the host require further investigation. To date, the clinical studies on ImP have generally been small in scale or conducted in specific populations. Accordingly, larger clinical studies in the future will contribute to verifying the universality and reliability of existing findings, as well as revealing the biological effects of ImP in different populations. Notably, there has of yet to be little consideration of the temporal changes in ImP levels, and consequently, longitudinal studies in different populations and disease contexts could enable us to establish the dynamic evolution of ImP levels during disease progression. Given the significant role of ImP in diabetes and related vascular complications, research on this metabolite spans multiple disease areas, and accordingly, multidisciplinary collaboration will facilitate further in-depth research.

At present, targeted therapeutic research on ImP remains limited, and a larger number of studies are needed to assess potential treatment strategies. For example, previous research has shown that CK and pirfenidone may inhibit the detrimental effects of ImP via different mechanisms, although these findings require further validation. Additionally, future research should also investigate the absorption, distribution, metabolism, and excretion (ADME) of ImP using isotopically labeled ImP to provide comprehensive insights into its pharmacokinetics. Developing other potential drugs or natural compounds and evaluating their efficacy and safety in inhibiting ImP activity will be a key focus for future studies. Additionally, research should also examine the role of dietary and lifestyle interventions in regulating ImP levels and alleviating related diseases. These efforts will contribute to developing new therapeutic strategies to improve the prognosis of those with ImP-related diseases.

As a microbial His-derived metabolite associated with diabetes, ImP may influence host metabolism and immune responses via multiple signaling pathways, thereby playing pivotal roles in the development of diseases such as diabetes, atherosclerosis, and heart failure. ImP serves not only as a potential biomarker for disease risk but also represents an important target for future therapeutic interventions. Future research should further examine the key enzymes and regulatory factors in the ImP metabolic pathway and its interactions with other metabolites. It will also be necessary to conduct larger-scale clinical studies to gain a more comprehensive understanding of the role of ImP in disease progression in different temporal and spatial contexts, populations, and disease backgrounds. Additionally, developing targeted interventions for ImP, including drugs, natural compounds, and dietary and lifestyle changes, and assessing their potential efficacy in the prevention and treatment of metabolic and cardiovascular diseases, is of the utmost importance.
